# Researches Concerning the Intermittent Fevers of the North of Africa

**Published:** 1839-07

**Authors:** 


### ART. III.

1. *Recherches sur les Fièvres Intermittentes du Nord de l'Afrique.* Par F. C. MAILLOT, D.M., Médecin des Salles Militaires de l'Hospice Civil de Douai.—*A Paris*, 1835. 8vo, pp. 47.

*Researches concerning the Intermittent Fevers of the North of Africa.* By F. C. MAILLOT, M.D., Physician of the Military Wards of the Civil Hospital of Douay.—*Paris*, 1835.

2. *Traité des Fièvres ou Irritations Cérébro-spinales Intermittentes, d'après des Observations receuillies en France, en Corse, et en Afrique.* Par F. C. MAILLOT, D.M., Ancien Médecin des Hôpitaux Militaires d'Ajaccio et d'Alger; ex-Médecin en chef de l'Hôpital Militaire de Bone.—*A Paris*, 1836. 8vo, pp. 420.

*Treatise on Intermittent Fevers or Cerebro-spinal Irritations, from Observations collected in France, Corsica, and Africa.* By F. C. MAILLOT, M.D., formerly Physician of the Military Hospitals of Ajaccio and Algiers, and ex-Physician in chief of the Military Hospital of Bona.—*Paris*, 1836.

3. *Beobachtungen und Untersuchungen über das Wechselfieber.* Von Dr. Karl Kremers, Bergarzt zu Pannesheide bei Aaachen.—*Aaachen*, 1837. 12mo, pp. 132.

*Observations and Enquiries on Intermittent Fever.* By Dr. K. Kremers, Physician to the Mines near Aix-la-Chapelle.—*Aachen*, 1837.

4. *Delle Malattie Periodiche e principalmente delle Periodiche Febbrili.* Saggio Critico di Pietro Manni, Professore di Medicina nell'Universita della Sapienza di Roma.—*Parigi*, 1837. 8vo. pp. 68.

*A Critical Essay on Periodical Maladies, and principally of Febrile Periodical Maladies.* By Pietro Manni, Professor of Medicine in the University Della Sapienza at Rome.—*Paris*, 1837.

The first of these publications consists of a memoir read to the Royal Academy of Medicine, founded upon observations made or collected by the author in the garrison of Bona, relative to the destructive epidemics of the years from 1832 to 1835. In those years, the garrison consisting of between three and four thousand men, 22,530 were admitted into the hospital, and 2513 died, or 1 in 8; or, according to the more particular statement, there were

| | | | | |
|---|---|---|---|---|
| Admitted in | 1832 . | 4033, of whom | 449 died; | or 1 in 7. |
| .. | 1833 . | 6704 .. | 1526 .. | 1 in $3\frac{1}{2}$. |
| .. | 1834 and 1835 . | 11593 .. | 538 .. | 1 in 20. |

M. Maillot's attention was first and principally directed to determining the analogies existing between the fevers of Bona and those which he had previously had an opportunity of observing at Algiers and in Corsica, with a view to deciding on the propriety of applying to them the treatment which he had found adapted to the latter. The establishment of the characteristic of intermittence, as common to both, seems to have been the result; the fevers of Bona, like those of Corsica and of Algiers, arising in the neighbourhood of marshes, the greater proximity of which to the troops at Bona gave a severer character to the fever; demanding, as it proved, a prompt and more energetic treatment. M. Maillot maintains the relation of the continued forms to the intermittent; and the tendency of the intermittent, if unchecked, to pass into the continued; and of the continued, if bleeding was employed, to pass into the intermittent or remittent. These circumstances, he says, convinced him that he had not to deal with true continued fevers, the gastro-enterites or gastro-cephalites of France. He concluded that the probability was that the affections before him were those spoken of by Torti, part of the character of which is "de intermittente sensim, acutam et malignam migrat;" and he resolved on giving the quinine boldly in all the continued cases, without waiting either for remissions or intermissions, which were "only instantaneous when they were obtained." The results, which are very striking, are seen in the diminished mortality exhibited in the above statement.

The intermittent fevers of the winter give place, with an increase of temperature, to severer forms, intermitting, remitting, and continued; and these circumstances mark, in M. Maillot's opinion, "the passage of the irritative congestions which accompany the attack to the gastro-enterites and gastro-cephalites which are so numerous in the hot season."

The remittent first replace the intermittent forms, and then give place to the continued. Those who have read the voluminous critical details appended by Dr. Boott to his Life of Dr. Armstrong, will know what a weight of evidence has been collected by that learned writer on these subjects. Torti ascribed the greater malignity of the summer fevers to the greater heat of the season; which, Dr. Boott adds, probably acts by increasing the excitability and irritability of the body, and giving intensity and diffusion to the remote cause. M. Maillot's opinion of the cause of the continued supervening on the remitting and intermitting forms being inflammation was also that of Torti; and was entertained by Dr. Armstrong; although its correctness may be open to doubt. That the intermittents prevailing in the south, either in spring or autumn, occasionally assume a highly malignant character, and constitute or pass into forms which are fatal in the third, fourth, or fifth period, and which are expressly denominated *pernicious*, is a fact well known to observers of disease in those regions. It may be observed, in relation to the probable influence of temperature, that the plague is described by Sydenham as appearing in England at the approach of summer, and declining at the approach of winter: and this he considered to belong to its inflammatory character. The same accurate observer remarks that, when autumnal intermittent fevers appear early (in England), as in July, they are not at first distinguishable from continued fevers, without a very strict examination; and that they only become more plainly intermittent later in the season. Nor has our own country, in former periods, been free from intermittent or remittent fevers of the most malignant and destructive kind. Up to the year 1729, these maladies were most severely prevalent; and connected often, as the southern fevers so notoriously are, with fatal dysentery.

In the Bona fever, M. Maillot says it was possible, as late as the first days of June, to cause a kind of remission of the symptoms. This remission, however, appears to have been no more than the morning remission of symptoms, so common in the continued fevers of our own climate; and even this was only induced after bloodletting; by which sometimes the fever was entirely subdued, and sometimes converted into a distinct intermittent: it often seems, on the other hand, to have run on to malignant and typhoid forms. And, at the end of June, the continued fevers were quite distinctly separated from the intermittent. Yet the continued fevers, it would seem, begin occasionally with a few intermittent paroxysms; after which they pursue their course even without remissions, however slight. The point of practice which M. Maillot is most anxious to enforce is, that, notwithstanding this appearance of continuity, the treatment demanded is the administration of bark in full doses. The same circumstances, and the necessity for this practice, were pointed out by M. Coutanceau in the epidemic pernicious fevers of Bordeaux, in 1805; and his opinions are quoted by M. Maillot, who declares them to be equally applicable to the fevers of Bona of 1832 and 1833. With these convictions, M. Maillot gave large doses of the sulphate of quinine in all the cases of continued fever, with the exception of some in which there was ileo-colitis; in which, although he thinks he was wrong in making them an exception, he deferred its administration. In all the cases thus treated, in which he enumerates cases of gastrocephalitis, of acute gastro-enteritis, of follicular ileo-colitis (diarrhœa), of hemorrhagic ileo-colitis (dysentery), &c. &c., the disease, except in a few instances, was relieved in a few days. In almost all these cases, the patients began to take some light food on the third or fourth day. Of ninety-eight cases of gastro-cephalitis included among them, occurring in the month of July, not one became typhoid; and only five died, of whom two sunk the day after admission into the hospital. In the other cases, the solution of the disease was speedy, and the convalescence rapid.

There is something surprising in this account, and we have allowed the reader to share our astonishment, although, at page 26 of his memoir, when M. Maillot comes to relate particular cases, we find another article of treatment generally preceding the use of the sulphate of quinine, and which, although it is no other than pretty free bleeding, general and local, had not been before alluded to as of the smallest importance.

"A soldier of the 59th, aged twenty-five years, was admitted into the hospital on the 8th of August, on the second day of an acute and excessively intense gastro-cephalitis. I immediately prescribed bleeding from the arm to fifteen ounces, the application of forty leeches to the epigastrium, and twenty leeches in the course of the jugulars; low diet; lemonade.

"On the 9th, at the morning visit, the reaction was not entirely subdued; but the condition of the pulse, that of the skin, and all the other symptoms, denoted a remission indicative of approaching remittence or intermittence; and I consider it a continued gastro-cephalitis, passing into intermittent or remittent fever.—Low diet; lemonade; twenty-four grains of sulphate of quinine to be taken in a potion at one dose, and immediately.

"Complete apyrexia established itself during the day. The apyrexia continued on the morning of the 10th: nevertheless, I prescribed another potion of twenty-four grains of sulphate of quinine, fearing that the fever might be tertian, and return the next morning. But the fever did not return; and convalescence went on rapidly. On the 18th, the patient was nearly on full diet." (p. 27.)

Such was very nearly the treatment of 295 cases of gastro-cephalitis; except that the sulphate of quinine, in the subsequent cases, was given immediately after the venesection; and, in certain circumstances, before any sanguine evacuation, as many of the men had been carried off by paroxysms of pernicious fever, some hours after the opening of a vein. Of the 295 cases thus treated, only twelve died, or 1 in 24. These results were certainly satisfactory: but M. Maillot observes, that such treatment would not be suitable to cases occurring in the north of France, in which dangerous typhoid affections, and (in case of recovery) tedious convalescence, would be the consequences. There can be little doubt that such would be the serious results of similar practice in the continued fevers of England; yet we believe there is much evidence of the most respectable kind among previous writers on the fevers of the Mediterranean, and of Italy, and of Africa, in support of the practice observed by M. Maillot.

Among the cases of true intermittent fever, 1582 were quotidian, 730 tertian, and 26 quartan. Of these 2338 cases of intermittent fever, the accession took place between midnight and noon in 1652, and between noon and midnight in 686. The greater number of accessions took place between nine in the morning and noon. 658 of the cases were simple, and 1680 complicated. In 1078 instances the intestinal canal was affected; alone in 343 cases: with the brain in 686 cases; with the lungs in 31 cases; with the brain and lungs in 13 cases. In 25 cases the spleen alone was diseased; and in one case the peritoneum alone. The brain was affected alone in 466 cases; the spinal cord in one; the lungs alone in 103 cases, and the pleura alone in five. In one case, a tertian, there was angina with the formation of a false membrane, and no other lesion. The intensity of all the complications was in direct ratio to the elevation of the temperature; and they were always unfavorably affected by the wind of the desert.

From the above observations, M. Maillot thinks himself authorized to conclude, that intermittent fevers are lesions of the nervous system in general, and especially of the cerebro-spinal axis. These lesions he considers to be hyperemiæ of the nervous centres; constituting, when local and slight, and uncomplicated with visceral irritation, simple intermittent fevers; but when intense and severe, the principal forms of pernicious fever; the *comatose* form, if the congestion is chiefly in the white central substance; the *delirious* form, if the membranes and the gray substance are affected; the *algide* form (with prolonged and icy coldness), if the congestion is established in the spinal cord. Death often takes place in all these forms, without any other viscus being sympathetically affected. The functional disorders of other organs, and their material affections, are only sympathetic, and are insufficient to produce the phenomena of intermittent fever. Dissection either discloses merely affections of the cerebro-spinal centre, or, if other viscera are affected, their diseased state is always complicated with lesion of the cerebro-spinal apparatus. The gastro-enteritis, or gastro-cephalitis are, in such cases, only subordinate irritative congestions, dependent on the return of the nervous phenomena, and do not constitute the intermittent fever.

In this country, pathologists have not been very ready to admit this theory of intermittent inflammatory conditions of the intestinal canal and brain. M. Maillot presents the doctrine in the least objectionable form. The secondary *congestions*, he says, are ordinarily very feeble in the first accessions, and disperse in the interval between one accession and the next. Complete apyrexia therefore ensues, without functional disorder of the digestive or respiratory passages. But when the accessions are many times repeated, and, above all, when they assume a quotidian type, each leaves some anatomical traces of congestion in the viscera affected. The capillaries soon become unable to disembarrass themselves of the blood which each accession determines to them; the tissues soon become unable to resist a state of congestion so frequently renewed, and the irritation "fixes itself anatomically," and betrays itself by symptoms more or less continued. Hence arises a prolongation of the reaction; that is to say, of the febrile symptoms, thirst, redness of the tongue, headach, heat of the skin, and all the symptoms of a gastro-enteritis, a gastro-cephalitis, a pneumonia, &c. according to the organs which are over-irritated (*surirrités*). (p. 36.)

To these remarks M. Maillot adds the very important practical observation, that simple irritations and those not of great intensity, and which yield in the intervals of an intermittent, give rise to symptoms in this class of fevers as marked and violent as those of acute gastro-cephalitis. This circumstance, he observes, if unknown or unattended to, might lead the practitioner to see inflammations where none exist, and to be afraid of administering the sulphate of quinine, on which alone the hope of preventing the returning accession of congestion must rest. In illustration of his practice, M. Maillot inserts a case in which, after bleeding during the paroxysm to fifteen ounces, the patient presenting the symptoms of acute gastro-cephalitis, twenty-four grains of sulphate of quinine were given at once, thirty leeches were applied to the epigastrium, and there was not another paroxysm. Of 250 cases thus treated, he only lost eleven, or 1 in 22: the fatal cases were all quotidian. In the pernicious forms of fever, with coma, he gave forty grains of the sulphate of quinine at a dose; and in one such case 148 grains were given in less than twenty hours, and the patient, from being in a state of coma, almost resembling death, became speedily and completely convalescent. In cases of the *algide* form, or with extreme coldness, ether was administered with the sulphate of quinine.

The most convincing proofs of the correctness of the above practice, and perhaps of the theory also, is that M. Maillot appears to have reduced the mortality in the fearful epidemic he had to contend with from 1 in 3½ to 1 in 20; for these results cannot be ascribed to any alteration in the character of the disorder; but became sensible when he began to use the sulphate of quinine more freely than he ventured to do at first, and to bleed less copiously. Subsequent engorgements of the abdominal viscera, dropsy, diarrhœa, so often considered to arise from the use of bark, were scarcely seen in any case; and M. Maillot considers them as the results not of the medicine, but of repeated paroxysms of the disease.

The title of M. Maillot's second and larger publication shows that further observation has made him sufficiently confident of the correctness of his views to apply the name of intermittent cerebro-spinal irritation to intermittent fevers. In this volume is of course collected much matter, illustrative of his views, and tending to show, by observations collected in different climates, that a high temperature imparts a peculiar character to intermittents, masking the apyrexia and periodicity, and giving an appearance of a continued type. The condition of the disease, he maintains, is an acute irritation, an hyperæmia of the great nervous centres.

The subject of the pernicious and anomalous forms of intermittents possesses much interest; and it is well and briefly treated in this publication. Affections to which the term *pernicious* has been applied are merely, M. Maillot observes (and as Broussais had before observed), cases characterized by the greater violence of the accompanying congestions; the danger depending on the importance of the organ affected. M. Maillot refers to the elucidation of these forms by Morton, Torti, Cleghorn, and others; and, putting aside the numerous and useless divisions of them made by Alibert, judiciously confines the term to those in which death is imminent or certain at the third or fourth accession, unless the malady is arrested. These forms depend, he maintains, on lesion of the cerebro-spinal apparatus, or of the abdominal organs, or of the thoracic viscera; a view of them which scarcely seems to admit of dispute.

Of the forms referrible to lesion of the cerebro-spinal apparatus, M. Maillot describes three, which are the most important: the comatose, the delirious, and the algide, or that icy-cold form for which we have no admitted English appellation.

In the *comatose* form, the stupor may vary in degree from simple oppression to profound carus. The pulse is full, large, without hardness, sometimes quickened, occasionally retarded; the respiration is slow, noisy, stertorous. The patient lies supine, and his limbs appear paralyzed; the jaw is firmly closed, and deglutition is difficult; sometimes there are epileptic spasms. These severe symptoms commonly occur with the second paroxysm, nothing taking place before to give warning of them, except it be some slowness of speech in the apyrexia. After an uncertain continuance of the comatose stage, the sweating stage follows, and the patient slowly recovers, wearing an extraordinary air of astonishment, and seeming to recover his senses one by one. Very full information concerning fevers of this kind is to be found in the excellent work of M. Bailly of Blois, published in 1825, and founded on observations chiefly made at the hospital of Saint Esprit at Rome, in 1820-21-22, to which we cannot do better than refer the reader, who is particularly interested in the nature and treatment of these dangerous affections, rare, or almost unknown, in our climate, but highly destructive in many localities where British practitioners in the army and navy are called upon to contend with them.

The *delirious* form of pernicious intermittent is, like the comatose, very common. Its name indicates its chief peculiarity. Death often takes place suddenly, without the supervention of coma: "life is broken by a single shock." If a salutary crisis ensues, the skin becomes moist and perspirable, the pulse loses its hardness, and the delirium gradually subsides.

The *algide* form is very peculiar. It is not, M. Maillot observes, at least generally, an indefinite prolongation of the cold stage. In the first stage of an intermittent, the sense of cold experienced by the patient is out of all proportion to the actual reduction of temperature; whereas in the algide fever, although the skin is icy-cold, the patient does not complain of coldness. And this cold state supervenes after reaction has commenced, and often suddenly. The circulation becomes disturbed, lowered, and the pulse can scarcely be felt, the temperature of the body at the same time rapidly decreasing. The extremities, the face, the trunk, become cold in succession, the abdomen remaining longer warm. The skin has the coldness of marble. The tongue becomes pale, moist, and cold, the lips are without colour, and the breath is cold. There is no thirst, and attempts to drink often excite vomiting. The actions of the heart become feeble, and only appreciable by auscultation. The intellectual faculties are undisturbed, and there is a sense of repose which is agreeable to the patient. All facial expression is lost. With this state, cholera may become conjoined, and the eyes then become hollow, glassy, and surrounded by a bluish circle. The approach of the algide form is so insidious as often to be mistaken for a remission produced by bloodletting, and the practitioner is only undeceived by the suddenness of the death of the patient. This deceitful calm is very strongly pointed out by M. Bailly, in his chapter on Diagnosis: he says that the patient may be walking about a few instants before his last attack; the accession is sudden, he lies down, and dies in a few hours.

Even when the pain (of the abdomen) and the danger are both considerable, the face has an appearance of calmness, as if its expression was no longer associated with the sufferings of other parts. Whenever, says M. Maillot, a sudden retardation of the pulse succeeds to reaction, and there is paleness of the tongue and discoloration of the lips, we should not hesitate to pronounce the case algide. Temporizing measures will be followed by death in a few hours. The patient dies as by an arrest of the innervation. If death does not take place, the pulse rises, the skin reacquires its natural warmth, and sometimes irritation of the brain or intestinal canal succeeds. Even this dangerous affection sometimes yields to remedial measures. The resemblance between this condition and cholera is commented upon by M. Maillot; and as, when left to itself, the algide form of fever is perhaps as fatal as cholera, he is of opinion that death in the latter affection has been too exclusively attributed to the excessive fluid evacuations.

These are the most general forms of pernicious intermittent. But sometimes the fatal symptoms are localized in the abdomen, constituting the gastralgic, choleric, dysenteric, and other forms. The *gastralgic* form is signalized by the acute, burning, tearing pain of the stomach; and in this state the face is contracted, and expressive of the utmost anxiety: this form is, however, seldom fatal. The *choleric* form is attended with very violent symptoms, which follow the periods of the fever, according to the expression of Torti, as the shadow follows the body. The *dysenteric* form, M. Maillot remarks, cannot with propriety be classed as pernicious, as when it is fatal, there is either chronic colitis, or the comatose, or delirious, or algide form of the disease has supervened. There are cases of intermittent fever in which jaundice takes place very suddenly, and disappears very slowly; this accident generally indicates a severe disease, to which the name of *icteric* fever has been applied.

To the forms in which sudden faintings occur during the attack, or palpitations, with pain at the heart and a feeble pulse, the names of *syncopal* and *carditic* have been given. When the lungs have been affected, the fever has been called *hemoptoic*, or *pleuritic*, or *pneumonic*, according to the complication.

Of all these forms of pernicious intermittent, M. Maillot gives examples; and he concludes that pernicious fevers only differ from ordinary intermittents by the violence of the congestions. They are most frequent in the hot seasons, when the visceral irritations which accompany the attacks are both more numerous and more intense.

In the chapters of M. Maillot's Treatise, in which illustrative cases are given, and in those on the causes, the mortality, the nature, and the diagnosis of intermittent affections, the reader will find much that will repay a careful perusal. His opinions on the subject of the treatment, founded on large experience, are all that we can find room to make the subject of present observation; and the importance of these is considerable: for, although the general principles on which an intermittent fever is to be treated, in whatever clime or season, must be the same, the indications always being to suspend the repetition of the attacks and to obviate their local effects, these indications require to be more promptly pursued in one locality than in another. The treatment adapted to the endemic of low and marshy places, may be unsuitable in a country that is higher and drier; and the means found to be successful in a temperate latitude may be inefficacious in a climate of higher temperature.

The remarkable cases published in the second edition of his work by the late lamented Dr. Mackintosh testify beyond doubt that the practice of bleeding in the cold stage of an intermittent has often been productive of immediate relief of the coldness, the sense of debility, and all the other phenomena of the cold stage, and has even appeared to cut short the disease. The relief was sometimes obtained after the loss of a few ounces; but the loss of twenty-four ounces was in other instances required. Several cases quoted from the Calcutta Transactions, or communicated by practitioners in India, afford similar evidence; and if M. Maillot's general plan of treatment had not been attended with results upon the whole so favorable to his practice, we might regret that he never ventured to give this method a trial. In the hot stage he generally bled to twelve or fifteen ounces, particularly when headach was present; and local bleedings were also practised when local symptoms pointed out their propriety: thirty or forty, or sometimes sixty or eighty leeches were applied to the epigastrium, or in the course of the jugular veins, or to the head, according to the circumstances of the case. M. Maillot thinks there is good reason for the opinion that the anterior lobes of the brain are more susceptible of inflammation than the other regions, and that leeches are consequently applied with most advantage to the forehead. The persistence of pain in the head, or marked disturbance of other organs, was considered an indication for repeating these measures. Obstinate vomiting was relieved by small portions of ice taken from time to time, and the diet was rigidly regulated. Of the different kinds of bark, M. Maillot prefers the red (*oblongifolia*), not only to the gray (*lancifolia*), which contains only cinchonia and not quina, but also to the yellow (*cordifolia*), from which, on account of the quantity of quina it contains, the disulphate of the London pharmacopœia is prepared. But he prefers the sulphate of quinine (disulphate of quina) to all preparations of bark; the doses being more easily regulated, the stomach tolerating it better, and its action being surer and speedier; qualities which practitioners are not all equally inclined, we think, to accord it the possession of. When the stomach rejected it, the sulphate was given in a lavement; if purging or colic ensued, the endermic method was had recourse to; and sometimes the patients were put into a bath saturated with cinchonia. In salicine and ilicine, M. Maillot places, as might be expected, little confidence. He seems not to have given any trials to opium; but he quotes the opinions of Lind and of M. Bailly to show that its power is only sedative, and not febrifuge; and that, if it seems to shorten the duration of the attacks, it does not prevent their recurrence. We have little doubt that there are cases in which opium mitigates the sufferings incidental to intermittents; and in some cases, at least in our own climate, a large dose given in the cold stage will put an end to it, and even to the disease altogether. M. Maillot agrees with M. Bailly in condemning the combination of antimony with opium as useless. With less reason he utterly condemns arsenical preparations. He also reprobates the notion of giving an emetic or even a purgative before commencing the administration of the sulphate of quinine; being of opinion that the attention of the practitioner, being directed to the subduing of abdominal or other

Orleans Parish Medical Society LIBRARY OF THE

irritations by leeches, would be uselessly given to removing any internal sources of irritation, which he looks upon as imaginary, and but the relics of exploded doctrines. In this respect, theory certainly interferes unfavorably with M. Maillot's practice; and that there are even cases of intermittent in which, after the application of leeches to the epigastrium, and the administration of a purgative, the disorder will disappear before a grain of bark is given, every one who has seen much of ague must have found. The general propriety of M. Maillot's rule, however, not to delay giving the sulphate of quinine as soon as a complete apyrexia is established, cannot be controverted; and the old doctrine of coction and crisis led without doubt to hurtful delay, during which the constitution suffered greatly from repeated paroxysms. He is, as we have seen, decidedly an advocate for giving the sulphate in doses of twenty or more grains, which he administers in four ounces of water; and the time he prefers is three or four hours before the expected attack. His experience in Africa determined him at length not to repeat the medicine more than once or twice after the suppression of a paroxysm; and he has pursued the same plan with success since his return to France. The prolonged use of the medicine is objectionable; and he agrees with M. Hepple in believing that it does not even prevent the return of the fever after a given time so certainly as having recourse to it anew, at the expected periods of return; supposing such periods to be ascertained, as stated by M. Hepple, namely, the eleventh and twenty-first day in quotidians and tertians, and between the twentieth and thirtieth in quartans.

The unfortunate tendency to a relapse makes it desirable, M. Maillot observes, that the patients should consider themselves as convalescents for two or three months, however well they may appear to be. When relapses take place, bleeding, except by leeches, is generally less required than at first; but the sulphate of quinine should be given in increased doses. In some cases a complete change of residence is indispensable to recovery; soldiers who were sent home to France after obstinate and renewed attacks often recovered health during the voyage.

The fevers of marshy countries during the hot-seasons, and the fevers of hot climates in all seasons, demand the application of all these parts of treatment with yet more promptitude and boldness. Care to remove the complications of these destructive fevers becomes absorbed in the intense anxiety of the practitioner to remove the malady itself, of which they form an essential part in such seasons or in such regions; and the treatment, less governed by the constitution of the patient or the period of the disease, becomes hardily empirical. In these circumstances we have seen that M. Maillot soon learned to administer the quinine without delay, and even to dispense with preparatory bloodletting. By the repetition of a violently congestive state, the intermittent fever had, he found, a tendency to become continued and to assume the pernicious form. These tendencies he combated by bleeding when the patient was first seen, either in the apyrexia or in any stage of reaction. If there remained headach in the interval, at least fifteen ounces of blood were taken away; and if the intestinal canal was irritated, leeches were applied to the epigastrium. If these symptoms were violent during the paroxysm, and still severe in the interval, twenty, or twenty-five, or thirty ounces of blood were taken at once.

These bleedings, which will not be considered so large by British practitioners as they seem to be in M. Maillot's estimation, were not so well borne by the patients in the great heats of July as in the spring; and sometimes the fevers put on a pernicious form afterward, an event to which M. Maillot attributes the prejudice against bleeding in these cases which exists in many countries, and especially in Italy. After the bleeding, M. Maillot insists strongly on the propriety, whatever the circumstances, of immediately giving the sulphate of quinine, in doses of from twenty-four to forty grains in a few ounces of water; after which all the symptoms will be found to disappear in a few hours, and, to use his own words, "as if by enchantment." Certainly, there is nothing more astonishing than the results of this kind of treatment appear to be in the various and fearful, and one would almost have said hopeless, cases, not only related by M. Maillot, but by Torti and other authors of authority on the fevers of hot climates. Combinations of symptoms, from which recovery would seem almost impossible, are dissipated with a rapidity of which in our climate we have no analogous examples. Some of the cases in M. Maillot's concluding chapter, are really marvellous, but at the same time, we are assured, perfectly worthy of belief. In one of them, an intense case of the comatose pernicious form of fever, 128 grains of sulphate of quinine were administered in a few hours. On the third day from the admission of this patient into the hospital, there was complete apyrexia, and on the fourth he was convalescent. The patient was also bled twice; and the importance of bleeding, both in the cases of comatose and delirious pernicious intermittent is strongly urged. A bleeding from the arm, followed by opening the temporal artery, was found to be extremely efficacious; and most so in the comatose form.

It appears, also, that blisters and sinapisms were applied in many cases; the first often preparatory to the introduction of the sulphate of quinine by the blistered surface, when, as in the choleric form, the medicine could neither be swallowed nor given by the rectum. In these forms, also, opium was sometimes conjoined with the quinine to the extent of ten grains in the course of the day.

The *algide* form is, as may be supposed from the description which has already been given of it, that in which the danger is the most imminent, and the necessity for vigorous practice the most urgent. M. Maillot gives an example in which forty grains of sulphate of quinine and two drachms of ether were given in four ounces of water, at two doses, in the course of an hour: a starch opiate injection, with sixty grains of the sulphate and two drachms of ether, was ordered at the same time; sinapisms to the legs, and a blister to each thigh. Under this sharp practice the patient began in a few hours to recover warmth, and the heart to act more forcibly; but the next morning the amendment was so slight, that a sinapism was applied to the whole length of the spinal column, and a lavement given with sixty grains of sulphate of quinine and three drachms of ether: strong reaction then took place, and recovery commenced.

Upon the whole, M. Maillot's book must be considered as a very interesting addition to our knowledge of the forms and treatment of the intermittent fevers of countries, the diseases of which have not yet, for want of sufficient opportunities, been fully observed. Any one who has looked into the discordant evidence, the violent controversies, the conflicting practical rules laid down by practitioners assuming to themselves the credit of experience in the fatal endemics of warm countries, must receive with curiosity at least, and with a somewhat grateful feeling, such details as those afforded by M. Maillot; and although some of his observations may be a little startling, the results of his practice, we repeat, are such as very strongly support his theoretical views. The work is written in a temperate spirit, and indicates both experience and habits of careful reflection.

Very little of M. Maillot's work is taken up with mere theoretical discussions. Alluding to some of the doctrines of intermittence, and particularly to that of M. Bailly, who ascribes it to the periodical alternations of the horizontal and erect positions in human beings, he prudently abstains from much personal speculation, either on this subject or on the mode of action of quina in subduing such a state. Dr. Manni's work has the air of an academical essay on questions of this kind, eloquently and diffusely written, but of no very direct application to practice. We may, however, mention Dr. Kremer's theory, leaving it for the reflection of the reader.

He asserts, that in all the intermittent fevers which, in his own neighbourhood (where they frequently occur), he has had an opportunity of observing, there exists at the situation of the first dorsal vertebra a more or less severe pain on pressure. In order to assure himself that this was not merely a characteristic of the particular epidemic during which he made his observation, he went to Antwerp, Brussels, Ghent, and Paris, where he saw many cases of intermittent fever, and met with sufficient evidence to confirm his previous experience.

In order to perceive the symptom, the pressure must be made from behind forwards, with the fingers upon the spinous process of the individual vertebra, not upon several vertebræ together; for in this latter case one bone is less affected in its relations with the others than in the former. If the intermittent fever is considerable, or old, or masked, pressure on the first dorsal vertebra, by giving pain, will suffice to evince the existence of fever. In simple or mild cases, the pain may be but trifling, and may require, particularly in phlegmatic individuals, that the attention shall be directed to it; and in order to render it evident, pressure should be first made on the first cervical vertebra, then on the last dorsal, and then lastly on the first dorsal, when the patient may be asked if he feels any difference in the sensations produced. The pain exists during the paroxysms, as well as in the apyretic interval, is stronger in epidemic than in sporadic intermittent fever, exists in all forms, and continues during the sequelæ.

We give this statement as we find it in Dr. Kremers' book, and with the view of calling the attention of our readers to corroborate or disprove it by their own observation. We confess that we are extremely sceptical as to its truth; and we find from the German journals, that some of the author's countrymen have not found the same results in examining cases of the disease, since the publication of his work. If it were established as a general truth, it would be important in a practical as well as a pathological point of view, as is remarked by the author. In practice, for instance, it would be a valuable diagnostic sign in cases of masked intermittent, and the visceral affections springing from such a cause. The author observes that we are entirely in want of a distinct diagnostic sign of the duration of intermittent fevers. A patient appears, for example, to be entirely cured of an autumnal ague; some few morbid phenomena occur during the winter, which appear to be quite unconnected with the fever, but which do not cease until after the occurrence of two paroxysms of a spring fever; or an intermittent fever returns during many successive years at a regular period, and does not come to an end until a gastric fever has run its tedious course.

Now, as the author observes, if the pain in the spine continues during the apyretic interval, it may also continue during this tedious period of indisposition, and may thus afford us a diagnostic sign of the constant connexion of apparently dissociated phenomena.

---

